# Improved detection of *esp, hyl, asa1, gelE, cylA* virulence genes among clinical isolates of Enterococci

**DOI:** 10.1186/s13104-020-05018-0

**Published:** 2020-03-20

**Authors:** Alexander Kiruthiga, Kesavaram Padmavathy, Praveen Shabana, Venkatesan Naveenkumar, Sumathi Gnanadesikan, Jeevan Malaiyan

**Affiliations:** 1 Department of Microbiology, Research Laboratory for Oral and Systemic Health, Sree Balaji Dental College and Hospital, BIHER, Velachery Main Road, Chennai, 600100 India; 2 Department of Microbiology, Priyadarshini Dental College and Hospital, Pandur, Thiruvallur, India; 3 Department of Laboratory Medicine, Meitra Hospital, Calicut, India; 4 ImmuGenix Biosciences Pvt Ltd, Chennai, India; 5 Department of Microbiology, Sri Muthukumaran Medical College Hospital and Research Institute, Chennai, India

**Keywords:** *E. faecalis*, *E. faecium*, Virulence, Modified PCR reactions

## Abstract

**Objective:**

Virulence factors (VFs) among the clinical strains of enterococci play a vital role in pathogenesis. This study was aimed to screen for *cylA, asa1, gelE, esp* and *hyl* among *Enterococcus faecalis* (n = 89) and *E. faecium* (n = 51) by multiplex PCR. The previously reported multiplex PCR was modified to 2 duplex (*asa1* and *gelE*, *cylA* and *esp*) PCRs and 1 simplex (*hyl*) PCR. The idea of the modification of the multiplex PCR proposed here emerged in the course of the research study when majority of the isolates which phenotypically exhibited virulence traits were found to be negative for the respective gene.

**Results:**

*cylA, gelE* and *asa1* were significantly predominant in *E. faecalis* (59.55%, 85.39%, 86.51%) than *E. faecium* (1.96%, 60.78%, 9.80%) (p < 0.0001, p = 0.001967, p < 0.0001). *hyl* was detected in *E. faecium* (5.9%) only. The number of VFs detected in each isolate was recorded as the VF score. *E. faecalis* isolates had a VF score pattern of score 4 (34.83%), score 3 (26.96%), score 2 (28.08%) and score 1 (8.98%) while *E. faecium* had score 4 (1.96%), score 3 (7.84%), score 2 (25.49%) and score 1 (41.18%). This modification of the PCR protocol could resolve the problem of decreased detection of virulence determinants in enterococci.

## Introduction

*Enterococcus faecalis* and *E. faecium,* the two most common species of enterococci that inhabit the gastrointestinal tract are a leading cause of opportunistic and nosocomial infections in humans. Pathogenesis of enterococci is attributed to an array of virulence factors (VFs) viz., aggregation substance (AS), gelatinase (Gel), cytolysin (Cyl), enterococcal surface protein (Esp) and hyaluronidase (Hyl).

Cytolysin elaborated by hemolytic strains of *E. faecalis* contributes to virulence in animal models and in human infections [1–3]. The cytolysin operon is a two-component system, lysin (L) encoded by *cylL1, cylL2, cylM*, *cylB* and an activator (A) encoded by *cylA* [4, 5]. Gelatinase encoded by *gelE*, is an extracellular zinc-endopeptidase/protease produced by *E. faecalis* that is capable of hydrolyzing gelatin, collagen, casein, hemoglobin, and other peptides [6]. Gelatinase production in *E. faecalis* contributes to virulence in animals and humans [7, 8]. Gelatinase damages the host tissue facilitating bacterial migration and spread [9], colonisation and persistence by biofilm formation [10]. Enterococcal surface protein, encoded by *esp*, is significantly higher among clinical isolates than faecal isolates and is associated with increased virulence [11], colonization and persistence in the urinary tract [12] and biofilm formation [13]. Aggregation substance, encoded by *asa1*, facilitates the conjugative transfer of sex pheromone gene-containing plasmids [14] and enhances virulence (adherence to renal tubular cells [15], heart endocardial cells [16] and internalization by intestinal epithelial cells [17]). Hyaluronidase, encoded by the chromosomal *hyl*, is reported to be specific for *E. faecium* [18, 19] and shows homology to the hyaluronidases of other Gram positive cocci [20]. Esp and Hyl are known to be specific for *E. faecium,* while AS, Gel, Cyl, Esp for *E. faecalis* [18, 19].

Though phenotypic and genotypic methods are available for the detection of VFs, majority of the previous studies (Additional file1: Table S1) have adopted the multiplex PCR protocol described by Vankerckhoven et al. [18]. Nevertheless, in our experience, we found isolates which phenotypically exhibited virulence traits were found to be negative for the respective gene. In addition, non-specific amplifications were observed. Hence, this study was designed with slight modifications to the existing multiplex PCR protocol [18].

## Main text

### Materials and methods

Clinical samples were collected after obtaining approval from the Institutional Ethical Committee, Sree Balaji Dental College & Hospital, BIHER, Chennai, India (IEC No: SBDCECM106/14/08/dt19.06.2014). A total of 140 clinical isolates of Enterococci (*E. faecalis* [n = 89], *E. faecium* [n = 51] from urine [n = 111], pus [n = 24], body fluids [n = 4] and blood [n = 1]) from patients with urinary tract infection, pyogenic wound infection, infected body fluids, bacteraemia attending tertiary care hospitals in Chennai, South India were included in the study. Species identification and characterization was performed as per standard biochemical tests [21] and further confirmed using Enterococcus Differential Agar supplemented with 1% 2,3,5-Triphenyl Tetrazolium Chloride (TTC) (HiMedia Laboratories Pvt Ltd, Mumbai, India).

#### Phenotypic screening of virulence

Hemolysin production was assessed using blood agar plates (5% defibrinated sheep blood). A clear zone of haemolysis around enterococcal colonies after incubation at 37 °C for 24 h was scored positive [22]. Gelatinase production was detected by stabbing enterococcal isolates into 12% gelatin and after incubation at 37 °C for 24 h, positive gelatinase activity was indicated by liquefied gelatin even after refrigeration at 4 °C for 4 h [22]. Slime production was detected using Congo Red agar. A positive slime layer formation was indicated by black pigmented enterococcal colonies after incubation at 37 °C for 24 h [23].

#### Multiplex PCR

DNA was extracted from overnight pure cultures of Enterococcal isolates by boiling lysis method. All Enterococci strains were screened for the presence of five VFs encoding genes (*asa1, cylA, esp, gelE* and *hyl*) using multiplex PCR as previously described [18]. Five primer pairs were used to amplify the genes *asa1, gelE* [18], *cylA* [24]*, esp* [25] and *hyl* [18]. All the primers used in the study were synthesised at Macrogen (South Korea). This multiplex PCR is the most commonly used protocol for screening of virulence genes among enterococci (Additional file 1: Table S1). However, in our study, a very low prevalence of the virulence determinants was detected. Isolates which phenotypically exhibited the virulence trait was found to be negative (gene not detected by the multiplex PCR protocol) for the respective gene. In addition, non-specific amplifications were observed, amplicon size were not specific to the one indicated in the reference article [18]. Hence, simplex PCR for the individual genes were performed, followed by PCR with all possible combinations of the 5 genes. Finally, the following combinations of PCR reactions were standardised.

#### PCR standardization

Three different PCR reactions were standardised [two duplex (*asa1* and *gelE*; *cylA* and *esp*) and one simplex (*hyl*) PCR]. Each 25 µl PCR reaction was set up with 2 µl of DNA template, 10× PCR buffer containing 15 mM MgCl_2_, 10 pmol of each primer specific for the respective gene (for duplex 1: *asa1*, *gelE,* duplex 2: *cylA*, *esp* and simplex: *hyl* (Macrogen, Korea), 0.5 U (for duplex 1: *asa1*, *gelE* and simplex: *hyl*) and 1U (for duplex 2: *cylA*, *esp*) of *Taq*DNA polymerase (Genet Bio Co, South Korea), 10 mM of each dNTP (BioBasic, Canada Inc) and 100 mM MgSO_4_ (New England BioLabs Inc, USA).

The cycling conditions include an initial denaturation at 95 °C for 5 min, followed by 30 cycles of denaturation (94 °C for 1 min), annealing (56 °C for 1 min), and extension (72 °C for 1 min), and a final extension for 8 min at 72 °C. PCR was carried out in Veriti™ 96-well Thermal Cycler, Applied Biosystem, USA. Known positive and negative controls were included for each run. DNA ladder, 100-bp (GeNet Bio, South Korea) was included as a molecular size marker.

#### DNA sequencing of virulence genes

PCR amplicons of each gene from representative isolates were purified by FavorPrep GEL/PCR Purification kit (Favorgen, Taiwan) and sequenced by Sanger sequencing method at Macrogen (South Korea) in single directions by respective forward primer using ABI PRISM^®^BigDye™ Terminator and ABI 3730XL sequencer (Applied Biosystems, USA). All the virulence gene sequences were compared with known sequences in NCBI Database by using BLAST analysis (http://www.ncbi.nlm.nih.gov/BLAST/) and the sequences were deposited in the NCBI GenBank database. (GenBank Accession numbers: MN398378 (*asa1*), MN398379 (*cylA*), MN398380 (*hyl*), MN398381 (*gelE*) and MN420464 (*esp*). These isolates were used as positive controls.

### Results

#### Virulence phenotype

Of the 140 isolates, 58 (41.4%) (*E. faecalis* (n = 53), *E. faecium* (n = 5)) isolates were found to be beta-hemolytic. All the beta-hemolytic *E. faecium* isolates were those isolated from urine. Gelatinase was produced by 55 (39.3%) isolates (*E. faecalis* (n = 33), *E. faecium* (n = 22)). Slime production was detected in 130 (92.8%) isolates (*E. faecalis* (n = 82), *E. faecium* (n = 48)).

#### Virulence genotype

Among the 5 virulence determinants screened, *asa1* were significantly more common in *E. faecalis* followed by *gelE,* while, *gelE* was the most common gene followed by *esp in E. faecium. cylA, gelE* and *asa1* were significantly more common in *E. faecalis* (59.55%, 85.39%, 86.51%) than *E. faecium* (1.96%, 60.78%, 9.80%) (p < 0.0001, p = 0.001967, p < 0.0001). *hyl* was detected only in *E. faecium* (5.9%) and not in *E. faecalis* (0%) (p = 0.0465). However, no difference was observed in the incidence of *esp* between species ((*E. faecalis* (53.93%) vs. *E. faecium* (45.09%), p = 0.406164).

#### Correlation between virulence phenotype and genotype

Among the 53 beta-hemolytic *E. faecalis* isolates, 48 (90.6%) harboured the *cylA* gene. Among the *E. faecalis* isolates, 33/33 (100%) gelatinase producers and 43/56 (77.8%) non-gelatinase producers harboured *gelE*. Among *E*. *faecium* isolates, 14/22 (63.6%) gelatinase producers and 17/29 (58.6%) non-gelatinase producers harboured the *gelE*. Among the *E. faecalis* (n = 82) isolates that were slime producers, 45 (54.9%) and 73 (89.02%) harboured *esp* and *asa1* while, 3 (42.9%), 4 (57.1%) of the non-slime producers (n = 7) possessed *esp* and *asa1* respectively. Among the *E*. *faecium isolates*, 22/48 (45.8%), 4/48 (8.3%) of the slime producers and 1/3 (33.3%), 1/3 (33.3%) of the non-slime producers harboured the genes, *esp* and *asa1* respectively (Table 1).

**Table 1:** Correlation between virulence phenotype and genotype

Virulence factors (encoding gene)	*E. faecalis* (n = 89)				*E. faecium* (n = 51)			
	P+ G+	P− G+	P+ G−	P− G−	P+ G+	P− G+	P+ G−	P− G−
Cytolysin (*cylA*)	48	5	5	31	0	1	5	45
Gelatinase (*gelE*)	33	43	0	13	14	17	8	12
Slime								
*asa1*	73	4	9	3	4	1	44	2
*esp*	45	3	37	4	22	1	26	2

P+ phenotypically expressed

P− phenotypically not expressed

G+ Gene detected

G− Gene not detected

#### Virulence score

Majority of the *E. faecalis* isolates causing UTI elaborated VFs compared to *E. faecium* (*p *< 0.0001, OR = 163.3333, 95% CI 20.2715–1316.0268) (Table 2). The number of VFs detected in each isolate was recorded as the VF score. Majority of the *E. faecalis* isolates had a VF score 4 (34.83%), followed by score 3 (26.96%), score 2 (28.08%), score 1 (8.98%) and score 0 (1.12%). Nevertheless, the VF score pattern exhibited by *E. faecium* was found to be in the reverse order: VF score 4 (1.96%), followed by score 3 (7.84%), score 2 (25.49%), score 1 (41.18%) and score 0 (23.53%) (Table 2). VF score 4 and 3 were quite common among *E. faecalis* than *E. faecium* (p < 0.0001, 0.0124) respectively. Nevertheless, VF score 1 was significantly associated with *E. faecium* (< 0.0001) (Table 3).

**Table 2:** Correlation of the virulence score of the enterococci with clinical source

Virulence factor score	*Species*	Urine (n = 111)	Pus (n = 24)	Blood (n = 1)	Fluid (n = 4)
VF score 5 (n = 0)	*E. faecalis (n *= *0)*	0	0	0	0
	*E. faecium* *(n *= *0)*	0	0	0	0
VF score 4 (n = 32)	*E. faecalis* (n = 31)	23	8	0	0
	*E. faecium* (n = 1)	1	0	0	0
VF score 3 (n = 28)	*E. faecalis* (n = 24)	20	2	1	1
	*E. faecium* (n = 4)	3	1	0	0
VF score 2 (n = 38)	*E. faecalis* (n = 25)	21	4	0	0
	*E. faecium* (n = 13)	8	4	0	1
VF score 1 (n = 29)	*E. faecalis* (n = 8)	6	2	0	0
	*E. faecium* (n = 21)	16	3	0	2
VF score 0 (n = 13)	*E. faecalis* (n = 1)	1	0	0	0
	*E. faecium* (n = 12)	12	0	0	0

**Table 3:** Comparison of VF scores between *E. faecalis vs E. faecium*

Virulence score	*E. faecalis* (n = 89)	*E. faecium* (n = 51)	*p* value	OR	95% CI
VF score 5 (n = 0)	0 (0%)	0 (0%)	1*	–	–
VF score 4 (n = 32)	31 (34.83%)	1 (1.96%)	< 0.0001*	26.7241	3.5205–202.8634
VF score 3 (n = 28)	24 (26.96%)	4 (7.84%)	0.0124*	4.3385	1.4112–13.3377
VF score 2 (n = 38)	25 (28.08%)	13 (25.49%)	0.888*	1.1418	0.5228–2.4939
VF score 1 (n = 29)	8 (8.98%)	21 (41.18%)	< 0.0001*	0.1411	0.0565–0.3525
VF score 0 (n = 13)	1 (1.12%)	12 (23.53%)	0.000*	0.0369	0.0046–0.294
VF score* Mean ± SD	2.854 ± 1.040	1.235 ± 0.971	< 0.0001**	–	–

* Mann–Whitney U test: comparison of VF scores between *E. faecalis* vs. *E. faecium*

** Student’s t test: comparison of 2 means

### Discussion

In our study, 39.3% of enterococci (*E. faecalis* (37.1%), *E. faecium* (43.1%)) were gelatinase producers. Our results are in concordance with previous Indian studies that have documented a lower incidence of gelatinase production in *Enterococci* [26, 27]. Recent studies have reported an incidence of *gelE* in the range of 31–91.4% (Additional file 1: Table S1). Our molecular studies indicated that *gelE* was the second most common (76.4%) VF detected in enterococci, more commonly in *E. faecalis* (85.39%) than *E. faecium* (60.78%). Among the *E. faecalis* studied, all the gelatinase producers (100%) harboured *gelE* gene while, the reverse was not true. In contrary, 63.6% of the gelatinase producing *E. faecium* isolates harboured *gelE* gene. In concordance with previous reports, *gelE* was present as a silent gene in *E. faecalis* (77.8%), and *E. faecium* (58.6%) [18, 28].

In line with previous reports, 41.43% of our enterococcal isolates were beta-hemolytic [26, 27, 29]. In our study, of the beta-hemolytic enterococci (41.43%), majority were *E. faecalis* (91.38%) while, only 8.62% were *E. faecium* isolates. Our results corroborate with previous reports that document a very low frequency of *cylA* among *E. faecium* compared to *E. faecalis* (Additional file 1: Table S1) Of note, all the beta-hemolytic *E. faecium* were urinary isolates that did not harbour the gene *cylA.* Nevertheless, majority (90.57%) of the beta-hemolytic *E. faecalis* (urine (71.7%), pus (16.98%), blood (1.89%)) harboured the *cylA* gene. This finding is of clinical significance as the frequency of death is five times higher in an enterococcal infection associated with cytolysin-producing strain compared to a non cytolysin-producing strain [30]. In our study, *cylA* was present as a silent gene in 13.88%, 2.17% of *E. faecalis* and *E. faecium* respectively.

Esp encoded by *esp* is associated with adhesion, colonisation and host immune evasion. Though previous reports suggest that *esp* is more common in *E. faecium*, in our study, incidence of *esp* was slightly higher in *E. faecalis* (53.93%) than *E. faecium* (45.09%) [31, 32]. The incidence of *esp* and *asa1* shows a wide variation in various reports (Additional file 1: Table S1). Among the slime producers, 54.9%, 89.02% of *E. faecalis* isolates, and 45.8%, 8.3% of *E*. *faecium* harboured *esp* and *asa1* respectively. In our study, *esp* and *asa1* were found to be silent genes in both *E*. *faecium* (33.3%, 33.3%) and *E*. *faecalis* isolates (42.9%, 57.1%). As reported earlier, *hyl* was detected only in *E. faecium* [33]. Nevertheless, a few studies have reported the incidence of *hyl* in both species [29, 34–39]. Significant difference was observed in the VF score between species. In line with previous studies, *E. faecalis* (61.8%) were found to be multi-virulent with VF Scores 4 or 3 while, VF score 1 was quite common *in E. faecium* (41.18%) [27, 36]. Majority of the urinary *E. faecalis* elaborated multiple VFs compared to *E. faecium.*

Non-expression of these virulence genes could be attributed to a triad of factors, (i) gene expression is triggered in late exponential phase at high cell densities, (ii) environmental factors might influence gene expression and (iii) in vitro phenotypic testing conditions are different from the in vivo conditions [28, 40]. Nevertheless, the presence of virulence determinants in the clinical isolates might contribute to increased severity as they could be expressed under optimum conditions in vivo. Metadata of the previous studies on the detection of virulence genes of enterococci by multiplex/duplex/simplex PCR is depicted in Additional file 1: Table S1 [18, 27, 29, 33–39, 41–59].

### Conclusion

We report that our simple modification of the existing multiplex PCR had increased the detection of the enterococcal virulence genes. Predominance of virulence genes was in order of *gelE* (76.43%) > *asa1* (58.57%) > *esp* (50.71%) > *cylA* (38.57%) > *hyl* (2.14%). Virulence determinants were more common in *E. faecalis* (*asa1* (86.51%), *gelE* (85.39%), *cylA* (59.55%)) than *E. faecium* (*asa1* (9.80%), *gelE* (60.78%), *cylA* (1.96%)). *hyl* was detected only in *E. faecium*. This modified PCR protocol could be useful to resolve the problem of decreased detection of virulence determinants in enterococci.

## Limitations of the study

This study lacks the analysis of other virulence factors elaborated by enterococci. Also, majority of the study isolates were from urine with very less number from other body fluids.

## Supplementary information

**Additional file 1.** Metadata.

## Data Availability

GenBank Accession numbers: MN398378 (*asa1*), MN398379 (*cylA*), MN398380 (*hyl*), MN398381 (*gelE*) and MN420464 (*esp*).

## Abbreviations

AS: Aggregation substance
Cyl: Cytolysin
Esp: Enterococcal surface protein
Gel: Gelatinase
Hyl: Hyaluronidase
PCR: Polymerase chain reaction
